# Confirmation of involvement of new variants at CDKN2A/B in pediatric acute lymphoblastic leukemia susceptibility in the Spanish population

**DOI:** 10.1371/journal.pone.0177421

**Published:** 2017-05-08

**Authors:** Angela Gutierrez-Camino, Idoia Martin-Guerrero, Nagore Garcia de Andoin, Ana Sastre, Ana Carbone Bañeres, Itziar Astigarraga, Aurora Navajas, Africa Garcia-Orad

**Affiliations:** 1 Department of Genetics, Physic Anthropology and Animal Physiology, University of the Basque Country, UPV/EHU, Leioa, Spain; 2 Department of Pediatrics, University Hospital Donostia, San Sebastian, Spain; 3 BioDonostia Health Research Institute, San Sebastian, Spain; 4 Department of Oncohematology, University Hospital La Paz, Madrid, Spain; 5 Department of Pediatrics, University Hospital Miguel Servet, Zaragoza, Spain; 6 Department of Pediatrics, University Hospital Cruces, Barakaldo, Spain; 7 BioCruces Health Research Institute, Barakaldo, Spain; German Cancer Research Center (DKFZ), GERMANY

## Abstract

The locus CDKN2A/B (9p21.3), which comprises the tumor suppressors genes *CDKN2A* and *CDKN2B* and the long noncoding RNA (lncRNA) known as *ANRIL* (or CDKN2B-AS), was associated with childhood acute lymphoblastic leukemia (ALL) susceptibility in several genome wide association studies (GWAS). However, the variants associated in the diverse studies were different. Recently, new and independent SNPs deregulating the locus function were also identified in association with ALL risk. This diversity in the results may be explained because different variants in each population could alter CDKN2A/B locus function through diverse mechanisms. Therefore, the aim of this study was to determine whether the annotated risk variants in the CDKN2A/B locus affect the susceptibility of B cell precursor ALL (B-ALL) in our Spanish population and explore if other SNPs altering additional regulatory mechanisms could be also involved. We analyzed the four SNPs proposed by GWAs and two additional SNPs in miRNA binding sites in 217 pediatric patients with B-ALL and 330 healthy controls. The SNPs rs2811712, rs3731249, rs3217992 and rs2811709 were associated with B-ALL susceptibility in our Spanish population. ALL subtypes analyses showed that rs2811712 was associated with B-hyperdiploid ALL. These results provide evidence for the influence of genetic variants at CDKN2A/B locus with the risk of developing B-ALL.

## Introduction

Acute lymphoblastic leukemia (ALL) is the most common childhood malignancy [1, 2]. The genetic basis of ALL susceptibility is broadly supported by its association with certain congenital disorders [3] and, more recently, by genome-wide association studies (GWAS). The two first GWAS independently identified three loci associated with childhood ALL susceptibility: 10q21.2 (*ARID5B*), 7p12.2 (*IKZF1*) [4, 5] and 14q11.2 (*CEBPE*) [5], results widely validated [3, 6–8]. Some of these loci were associated with specific genetic subtypes of ALL, such as locus 10q21.2 (*ARID5B*) and B-hyperdiploid ALL [4, 5]. Subsequent GWAS discovered additional susceptibility loci at 10p12.2 (*BMI1-PIP4K2A*) [3], validated in some populations [9], but not in others [10], and 9p21.3 (*CDKN2A/B*)[11], a region that comprises the tumor suppressors genes *CDKN2A* and *CDKN2B* and a long noncoding RNA (lncRNA) known as *ANRIL*.

The region 9p21.3 is particularly noteworthy because independent association signals have been recently discovered at this locus in association with B cell precursor ALL (B-ALL) susceptibility. The first variant identified in children from the United Kingdom in 2010 was rs3731217 [11], which is located in intron 1 of *CDKN2A*. This association was replicated in several populations such as Germany, Canada [11] and France [12], but not in others like Poland [13], Hispanic [14] or Thai population [8]. In 2012, Orsi et al. [12] also associated one variant located in intron 1 of *CDKN2A*, rs2811709, with B-ALL in French children, a variant in low linkage disequilibrium (LD) with rs3731217 (r^2^<0.8). Then, in 2015, three independent studies using genotyping and imputation-based fine-mapping, pointed to rs3731249 in exon 2 of *CDKN2A* as the hit associated variant that conferred high risk for B-ALL in European and Hispanic children [15–17]. Finally, in 2016, Hungate et al., pointed to rs662463 in *ANRIL* as an independent locus associated with B-ALL susceptibility in European and African-Americans [18].

These variants could alter the locus through diverse mechanisms. The alleles of rs3731217 create two overlapping cis-acting intronic splice enhancer motifs (CCCAG**G** and CAG**T**AC) that may regulate alternative splicing of *CDKN2A* [18]. The SNP rs3731249 is a missense SNP in *CDKN2A* which produces an alanine-to-threonine change in amino-acid-sequence, resulting in reduced tumor suppressor function of p16^INK4A^ [15]. Interestingly, this SNP is also located in the 3´UTR region of p14^ARF^, where it creates a binding site for miR-132-5p and miR-4642 [19] and could cause the downregulation of the locus. More than other 40 SNPs in 3´UTR region of *CDKN2A* and *CDKN2B* that disrupt or create microRNA (miRNA) binding sites have been described, but studies focused on SNPs in miRNA binding sites are almost absent. Finally, rs662463 in *ANRIL* regulates *CDKN2B* expression by disrupting a transcription factor binding site for CEBPB [18].

Therefore, although there is an obvious implication of CDKN2A/B locus in B-ALL susceptibility, the variants annotated by the different studies are different and independent. This may be due to the fact that different variants in each population could alter CDKN2A/B locus function through diverse mechanisms. Therefore, the aim of this study was to determine the involvement of these variants at CDKN2A/B locus in the susceptibility of B-ALL in our Spanish population and explore if SNPs in miRNA binding sites could be also involved in B-ALL risk.

## Materials and methods

### Ethics statement

The study was approved by the local ethics committee CEIC-E (PI2014039) and was carried out according to the Declaration of Helsinki. Written informed consent was obtained from all participants, or from their parents, prior to sample collection.

### Study participants

A total of 231 European descent children diagnosed with B-ALL between 2000 and 2011 in the Pediatric Oncology Units of four Spanish hospitals (University Hospital Cruces, University Hospital Donostia, University Hospital La Paz and University Hospital Miguel Servet) and 338 unrelated healthy controls were included in this study (Table 1 and S1 Table). This is the sample size approximately needed to obtain a statistical power of 80% in a two sided χ2 test given a significance level of p = 0.05, 1.5 controls per case, a minor allele frequency (MAF) of 10% in the control group, and an Odd Ratio (OR) of approximately 2 [20].

**Table 1:** Patient characteristics and genetic alterations in the study population.

	Patients	Controls
**No. of individuals**	231	338
**Mean age ± SE, y**	4.04 ± 3.61	57.8 ± 28.1
**Age range, y**	1–16	21–101
**Sex**^a^		
**Males, n (%)**	128 (55.7)	157 (46.4)
**Females, n (%)**	102 (44.3)	181 (53.6)
**Genetic alterations**^b^		
**Hyperdiploid**	56 (24.2)	-
***ETV6-RUNX1***	37 (16.0)	-
***MLL***	13 (5.6)	-
***BCR-ABL***	6 (2.6)	-
***E2A-PBX1***	6 (2.6)	-
**Hypodiploid**	2 (0.9)	-
**Other**	1 (0.4)	-
**No alteration**	95 (41.1)	-
**Not available**	21 (9.1)	-

SE: standard error, y: years

^a^ There is no data for one patient.

^b^Six patients have more than one alteration

Data were collected objectively, blinded to genotypes, from the patients’ medical files. The two most common ALL subtypes, B-lineage hyperdiploid ALL with more than 50 chromosomes (B-hyperdiploid ALL) and B-lineage ALL bearing the t(12;21)(p13;q22) translocation leading to an *ETV6-RUNX1* gene fusion, were also analyzed. The other subtypes were not considered due to the low number of patients in our cohort. Sex and age data were systematically recorded (Table 1).

### Selection of polymorphisms

A total of six SNPs at the locus 9p21.3 were selected (S2 Table). Selection was done based on the following criteria: “(i) four SNPs previously reported to be highly associated with ALL susceptibility in the literature. Due to design options, for some of them we selected SNPs in high LD defined using the International HapMap Project (release #24; http://hapmap.ncbi.nlm.nih.gov/) (The HapMap Data Coordination Center (DCC), Bethesda, MD) and Haploview software v.4.2 (http://www.broad.mit.edu/mpg/haploview/) (Broad Institute, Cambridge, USA) with an r^2^ threshold of 0.8. (ii) SNPs in miRNA binding sites of 3´UTR region of *CDKN2A* and *CDKN2B* with a MAF>10% identified using bioinformatics tools: Ensembl (http://www.ensembl.org/) (Welcome Trust Genome Campus, Cambridge, UK), and miRNASNP (http://bioinfo.life.hust.edu.cn/miRNASNP2/index.php) (College of Life Science and Technology, HUST). Of 47 SNPs identified in the 3´UTR region that disrupt or create miRNA binding sites (S3 Table), only two had a MAF>10%.

### Genotype analyses

Genomic DNA was extracted from remission peripheral blood or bone marrow using the phenol-chloroform method as previously described [21]. DNA was quantified using PicoGreen (Invitrogen Corp., Carlsbad, CA).

For each sample, 400 ng of DNA were genotyped using the GoldenGate Genotyping Assay with Veracode technology according to the published Illumina protocol. Data were analyzed with GenomeStudio software for genotype clustering and calling. Duplicate samples and CEPH trios (Coriell Cell Repository, Camden, NJ) were genotyped across the plates. For rs3731249, the genotyping analyses were performed by using PCR followed by restriction analysis with *BstUI* enzyme. Duplicates were included in each assay. The PCR products were visualized after electrophoresis on 3% agarose gels. Primer sequences and PCR conditions are described in detail in S4 Table.

### Statistical analysis

To identify any deviation in Hardy-Weinberg equilibrium (HWE) for the healthy controls, a χ2 test was used. The association between genetic polymorphisms in cases and controls, as well as ALL subtypes and controls, was also evaluated using the χ2 or Fisher’s exact test. The effect sizes of the associations were estimated by the odds ratio from univariate logistic regression. The most significant test among codominant, dominant, recessive, and additive genetic models was selected. The results were adjusted for multiple comparisons using the false discovery rate (FDR)[22]. In all cases, the significance level was set at 5%.

## Results

### Genotyping results

A total of 231 patients with B-ALL and 338 unrelated healthy controls were available for genotyping with GoldenGate Genotyping Assay. Successful genotyping was achieved for 217 patients with B-ALL and 330 controls (96.1%). Of the SNPs, 5/5 (rs2811712, rs3217992, rs2811709, rs3731222 and rs1063192) were genotyped satisfactorily (95.6%, 95.3%, 85.4%, 95.3% and 95.6%, respectively). For rs3731249, 180 patients with B-ALL and 235 controls were available for genotyping with a genotyping rate of 97.6%. All of them were in HWE in the control cohort.

### Genotype association study of B-ALL

Of the 6 SNPs analyzed, we found 4 significantly associated with B-ALL risk (Table 2 and S1 Fig). From them, rs2811712 at *CDKN2B* displayed the most significant value under the log-additive genetic model (AA vs AG vs GG). The GG genotype showed a 1.98-fold increased risk of B-ALL (95% CI: 1.39–2.82; P = 0.0001). The second most significant association signal was found for rs3731249 at *CDKN2A*. In this case, the CT/TT genotypes produced a 2.61-fold increased risk of B-ALL (95% CI: 1.38–4.92; P = 0.002). We also found AA genotype of rs3217992 associated with a decreased risk of B-ALL (OR: 0.56; 95% CI: 0.36–0.88; P = 0.009). Finally, rs2811709 AG/AA genotypes were associated with a 1.7-fold increased risk of B-ALL. All the SNPs remained statistically associated with B-ALL risk after FDR correction. The SNPs rs3731222 and rs1063192 were not associated with B-ALL susceptibility in our population.

**Table 2:** Association results of SNPs in CDKN2A/B and B-ALL.

Gene	Genotype	N (controls)	N(cases)	OR (CI 95%)	P
SNP		(N = 330)	(N = 217)		
***ANRIL***	AA	264 (80.2)	143 (66.5)	Additive	0.0001 ^a^
**rs2811712**	AG	62 (18.8)	64 (29.8)	1.98 (1.39–2.82)	(0.0006)
	GG	3 (0.9)	8 (3.7)		
	A	590 (89.7)	350 (81.4)	1.98 (1.39–2.81)	0.0001 ^a^
	G	68 (10.3)	80 (18.6)		(0.0006)
***CDKN2A***	CC	217 (92.7)	142 (83)	Dominant	0.002 ^a^
**rs3731249**	CT	16 (6.8)	28 (16.4)	2.61 (1.38–4.92)	(0.006)
	TT	1 (0.4)	1 (0.6)		
	C	450 (96.2)	312 (91.2)	2.4 (1.31–4.38)	0.004 ^a^
	T	18 (3.8)	30 (8.8)		(0.012)
***CDKN2B*, *ANRIL***	GG	95 (28.9)	72 (33.8)	Recessive	0.009 ^a^
**rs3217992**	AG	153 (46.5)	108 (50.7)	0.56 (0.36–0.88)	(0.018)
	AA	81 (24.6)	33 (15.5)		
	G	343 (52.1)	252 (59.2)	0.75 (0.58–0.96)	0.023 ^a^
	A	315 (47.9)	174 (40.8)		(0.034)
***CDKN2A***	GG	203 (79.6)	145 (69.7)	Dominant	0.014 ^a^
**rs2811709**	AG	49 (19.2)	59 (28.4)	1.7 (1.11–2.59)	(0.021)
	AA	3 (1.2)	4 (1.9)		
	G	455 (89.2)	349 (83.9)	1.58 (1.08–2.32)	0.017 ^a^
	A	55 (10.8)	67 (16.1)		(0.034)
***CDKN2A***	AA	246 (74.8)	165 (77.5)	Dominant	0.47
**rs3731222**	AG	78 (23.7)	44 (20.7)	0.86 (0.57–1.29)	
	GG	5 (1.5)	4 (1.9)		
	A	570 (86.6)	374 (87.8)	0.9 (0.62–1.29)	0.57
	G	88 (13.4)	52 (12.2)		
***CDKN2B*, *ANRIL***	TT	125 (38.1)	86 (39.8)	Dominant	0.68
**rs1063192**	CT	162 (49.4)	98 (45.4)	0.93 (0.65–1.32)	
	CC	41 (12.5)	32 (14.8)		
	T	412 (62.8)	270 (62.5)	1.01 (0.78–1.3)	0.91
	C	244 (37.2)	162 (37.5)		

Abbreviations: CI, confidence interval; OR, odds ratio; SNP, single-nucleotide polymorphism

^a^ Significant after FDR correction, the p value is displayed in brackets

### Genotype association study of B-ALL subtypes

When we analyzed the 6 SNPs considering B-hyperdiploid ALL and *ETV-RUNX1* ALL subtype, we found association between TT genotype of rs3731249 and B-hyperdiploid ALL (OR:2.62; 95% CI:1.06–6.48; P = 0.048), AA genotype of rs3217992 with *ETV6*-*RUNX1* ALL (OR:0.58; 95% CI:0.34–0.96; P = 0.03) and GG genotype of rs2811712 with both B-hyperdiploid ALL (OR:8.69; 95% CI:1.89–40.0; P = 0.007) and *ETV6*-*RUNX1* ALL (OR:2.4; 95% CI:1.15–5.02; P = 0.024) (Table 3). After FDR correction, the association between rs2811712 and B-hyperdiploid ALL remained statistically significant (p = 0.042).

**Table 3:** Association results of SNPs in CDKN2A/B and B-hyperdiploid ALL and *ETV6-RUNX1* ALL.

			B-hyperdiploid ALL			*ETV6-RUNX1* ALL		
Gene	Genotype	N (controls)	N (cases)	OR (CI 95%)	P	N (cases)	OR (CI 95%)	P
SNP		(N = 330)	(N = 54)			(N = 37)		
***ANRIL***	AA	264 (80.2)	40 (74.1)	Recessive	0.007^a^	22 (62.9)	Dominant	0.024
**rs2811712**	AG	62 (18.8)	10 (18.5)	8.69 (1.89–40.0)	(0.048)	12 (34.3)	2.4 (1.15–5.02)	
	GG	3 (0.9)	4 (7.4)			1 (2.9)		
	A	590 (91.2)	90 (90.7)	1.73 (0.98–3.05)	0.055	56 (80)	2.16 (1.14–4.1)	0.017
	G	68 (8.8)	18 (9.3)			14 (20)		
***CDKN2A***	CC	217 (92.7)	39 (83)	Dominant	0.048	28 (90.3)	Dominant	0.64
**rs3731249**	CT	16 (6.8)	8 (17)	2.62 (1.06–6.48)		3 (9.7)	1.37 (0.38–4.96)	
	TT	1 (0.4)	0			0		
	C	450 (96.2)	86 (91.5)	2.32 (0.98–5.51)	0.55	59 (95.2)	1.27 (0.36–4.44)	0.70
	T	18 (3.8)	8 (8.5)			3 (4.8)		
***CDKN2A***	GG	203 (79.6)	38 (70.4)	Additive	0.06	23 (67.6)	Dominant	0.12
**rs2811709**	AG	49 (19.2)	13 (24.1)	1.72 (0.98–3.01)		11 (32.4)	1.87 (0.86–4.07)	
	AA	3 (1.2)	3 (5.6)			0		
	G	455 (89.2)	89 (82.4)	1.76 (0.99–3.11)	0.05	57 (83.8)	1.59 (0.79–3.22)	0.19
	A	55 (10.8)	19 (17.6)			11 (16.2)		
***CDKN2B*, *ANRIL***	TT	125 (38.1)	23 (42.6)	Dominant	0.53	12 (34.3)	Dominant	0.65
**rs1063192**	CT	162 (49.4)	24 (44.4)	0.83 (0.46–1.49)		17 (48.6)	1.18 (0.57–2.46)	
	CC	41 (12.5)	7 (13)			6 (17.1)		
	T	412 (62.8)	70 (64.8)	0.91 (0.59–1.4)	0.68	41 (58.8)	1.19 (0.72–1.97)	0.48
	C	244 (37.2)	38 (35.2)			29 (41.4)		
***CDKN2A***	AA	246 (74.8)	41 (77.4)	Dominant	0.68	27 (77.1)	Dominant	0.75
**rs3731222**	AG	78 (23.7)	12 (22.6)	0.87 (0.44–1.73)		7 (20)	0.88 (0.38–2.01)	
	GG	5 (1.5)	0			1 (2.9)		
	A	570 (86.6)	94 (88.7)	0.82 (0.43–1.57)	0.56	61 (87.1)	0.95 (0.45–1.99)	0.90
	G	88 (13.4)	12 (11.3)			9 (12.9)		
***CDKN2B*, *ANRIL***	GG	95 (28.9)	17 (31.5)	Recessive	0.93	15 (44.1)	Aditive	0.030
**rs3217992**	AG	153 (46.5)	24 (44.4)	0.97 (0.5–1.9)		15 (44.1)	0.58 (0.34–0.96)	
	AA	81 (24.6)	13 (24.1)			4 (11.8)		
	G	343 (52.1)	58 (53.7)	0.93 (0.62–1.41)	0.76	45 (66.2)	0.55 (0.32–0.94)	0.028
	A	315 (47.9)	50 (46.3)			23 (33.8)		

Abbreviations: CI, confidence interval; OR, odds ratio; SNP, single-nucleotide polymorphism

^a^ Significant after FDR correction, the p value is displayed in brackets

## Discussion

In the current study, we analyzed 6 SNPs at the CDKN2A/B locus in 217 children with B-ALL and 330 controls in a Spanish cohort. SNPs rs2811712, rs3731249, rs3217992 and rs2811709 were associated with B-ALL susceptibility. In the subtype analysis, rs2811712 was associated with the risk of developing B-hyperdiploid ALL.

The most significant finding was the association between GG genotype of rs2811712 and the increased risk of developing B-ALL (p = 10^−4^). This result is in line with most of the studies [9, 11, 14, 18]. The SNP rs2811712 is in strong LD with two of the previously reported SNPs, rs17756311 (r^2^ = 0.83) and rs662463 (r^2^ = 1), both associated with B-ALL risk in European Americans [3, 18] and African Americans [18], respectively. In the subtype analysis, GG genotype of rs2811712 was also associated with B-hyperdiploid ALL after FDR correction, result that is in line with Chokkalingam et al ‘study performed in Hispanics [14]. These associations could be explained considering that rs2811712 is located in intron 1 of the lncRNA *ANRIL*. SNPs in lncRNAs may affect its expression or its structure by interfering with lncRNA folding or by modulating protein-lncRNA interactions [23]. *ANRIL* has been shown to regulate *CDKN2A* and *CDKN2B* genes. Specifically, acting in cis, ANRIL binds various Polycomb proteins resulting in histone modification of the *CDKN2A/CDKN2B* locus, and in turn, silencing the cluster [24]. In fact, the G allele of rs2811712 was shown to decrease CDKN2B mRNA levels [25]. Therefore, the G allele of rs2811712 in *ANRIL* could be involved in the downregulation of the locus, contributing to increased susceptibility to B-ALL.

The second most significant association was found for the CT/TT genotypes of rs3731249, which produced a 2.6-fold increased risk of B-ALL (P = 0.002). This association was also described recently by 3 independent studies, all of them pointing out the high impact of this variant, since it confers in all studies between two and three-fold increased risk of B-ALL susceptibility in children of European and Hispanic origin [15–17]. Rs3731249 localizes to exon 2 of *CDKN2A*, being shared by both p16^INK4A^ and p14^ARF^, the tumor suppressors codified by *CDKN2A*. For the p16^INK4A^, the C-to-T nucleotide substitution resulted in an alanine-to-threonine change (p.A148T). There is evidence that the variant p16^INK4A^ (p.148T) is preferentially retained in the nucleus, compromising its ability to inhibit CDK4 and CDK6 in the cytoplasm [15] and favoring proliferation, and therefore contributing to the association with ALL risk. In p14^ARF^, rs3731249 is in the 3´UTR region, where the risk allele creates a miRNA binding site for miR-132-5p and miR-4642 [19]. These miRNAs could downregulate p14^ARF^ expression, and then, attenuate its function as cyclin inhibitor. Therefore, T allele of rs3731249 in *CDKN2A* could be involved in B-ALL through its effect on the function of both p16^INK4A^ and p14^ARF^.

The third finding was the association between the AA genotype of rs3217992 and a decreased B-ALL risk. This SNP is located in a miRNA binding site in *CDKN2B* in which the A allele disrupts the binding for miR-138 and miR-205 [26]. The loss of binding of these miRNAs could increase the expression of the tumor suppressor *CDKN2B*, explaining its protective role. As far as we know, this is the first time that this SNP is associated with B-ALL risk.

Regarding rs2811709, AG/AA genotypes were associated with an increased risk of B-ALL susceptibility in our population. This SNP was also associated with B-ALL risk in two previous studies of children of European origin [11, 12]. rs2811709 is a cis-eQTL for *CDKN2B*, with a decreased expression of CDKN2B mRNA for the risk allele [25], which could describe the involvement of rs2811709 in B-ALL.

On the other hand, we found no association between rs3731222 and rs1063192 and B-ALL susceptibility. One of them, rs3731222, is in high LD with rs3731217 (r^2^ = 1), the SNP identified in the work performed by Sherborne et al. [11] and replicated in several studies [9, 12]. However, we and others could not replicate this association [8, 13]. This lack of replication could be due to differences in the variants that are involved in the disease in the different populations.

Finally, this study has some limitations that might be addressed, such as the small sample size compared to other replication studies (13, 14). However, when we calculated the sample size needed to obtain an 80% of statistical power using the software OpenEpi [20] the estimated size was similar to our cohort. Another limitation could be that the putative function of miRNA binding site disruption was predicted by *in silico* tools, but nowadays, the possible inaccuracy of the prediction algorithms of the databases used has to be assumed.

In conclusion, three of the variants previously proposed by the literature, rs2811712, rs3731249 and rs2811709, and a new variant, rs3217992, were associated with B-ALL susceptibility in our Spanish cohort. These results confirmed the implication of CDKN2A/B locus in the development of B-ALL since all these SNPs could act through different mechanisms that might alter the cluster. The identification of the specific causes that could lead to the development of B-ALL is clearly a worthwhile goal for prevention or early intervention of this disease.

## Supporting information

S1 Fig Diagram of CDKN2A/B locus. In bold, the SNPs significantly associated with B-ALL risk in our study.
(PDF)

S1 Table Original data of all included patients. (XLSX)

S2 Table Selection of SNPs. (PDF)

S3 Table SNPs identified in 3´UTR region of *CDKN2A* and *CDKN2B*. SNPs with a MAF>10% are in bold.
(PDF)

S4 Table Primers and PCR conditions for the amplification of rs3731249 in *CDKN2A*. SNPs, single nucleotide polymorphisms; PCR, polymerase chain reaction; RFLP, restriction fragment length polymorphism; bp, base pairs.
(PDF)
