# Supplementary figures and images for: Confirmation of involvement of new variants at CDKN2A/B in pediatric acute lymphoblastic leukemia susceptibility in the Spanish population
Source: PLoS One. 2017 May 8;12(5):e0177421. doi: 10.1371/journal.pone.0177421 (PMC5421813; doi:10.1371/journal.pone.0177421)

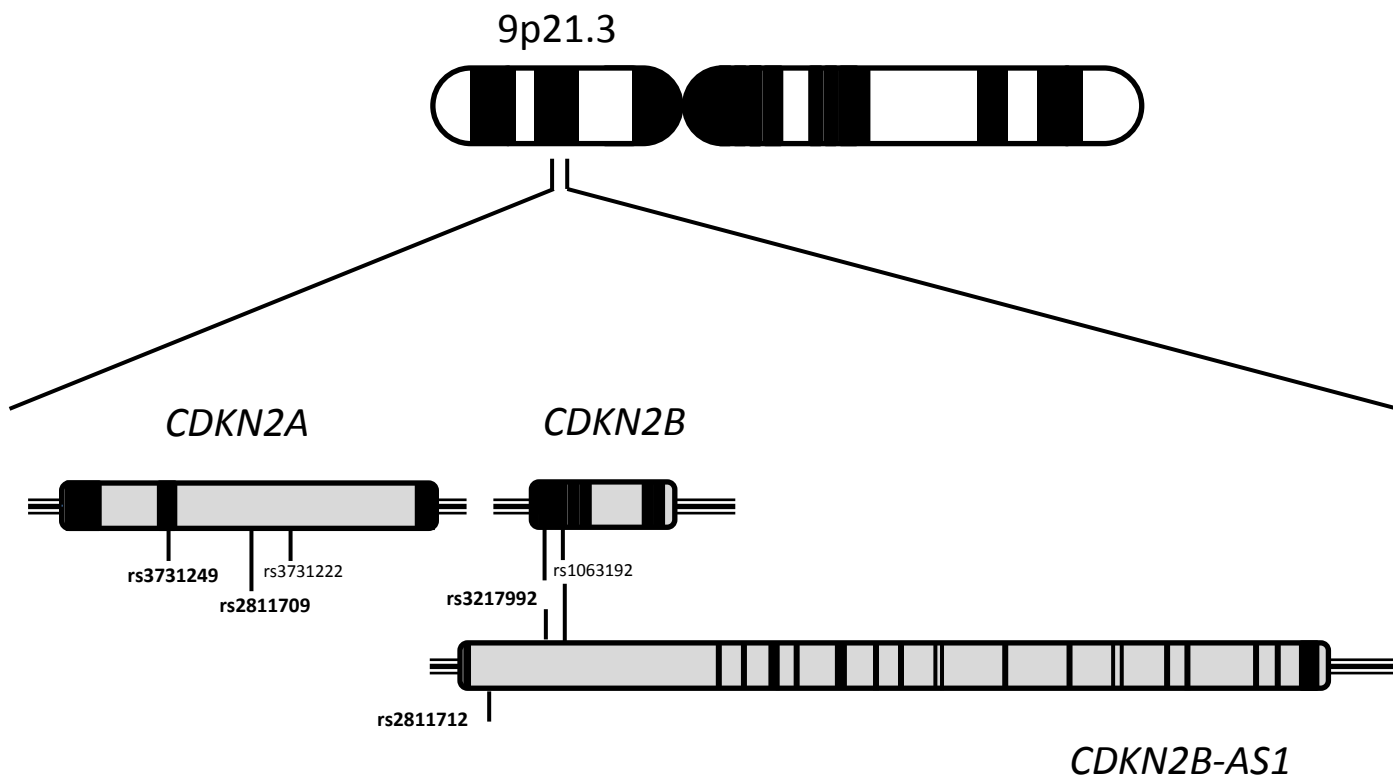

Supplement: S1 Fig — In bold, the SNPs significantly associated with B-ALL risk in our study. (PDF) [file pone.0177421.s001.pdf]
