# Supplementary material for: Confirmation of involvement of new variants at CDKN2A/B in pediatric acute lymphoblastic leukemia susceptibility in the Spanish population
Source: PLoS One. 2017 May 8;12(5):e0177421. doi: 10.1371/journal.pone.0177421 (PMC5421813; doi:10.1371/journal.pone.0177421)
Supplement: S2 Table — (PDF) [file pone.0177421.s003.pdf]

S2 Table: Selection of SNPs

SNP	Gene	Alleles	Reason for selection
**rs3731222**	*CDKN2A*	A>G	In LD with rs3731217 (Sherborne 2010)
**rs2811709**	*CDKN2A*	G>A	Bibliography (Orsi 2012)
**rs2811712**	*ANRIL*	A>G	In LD with rs662463 and rs17756311(Xu 2013, Hungate 2016)
**rs3731249**	*CDKN2A*	C>T	Bibliography (Xu 2015, Walsh 2015, Vijayakrishnan 2015)
**rs1063192**	*CDKN2B, ANRIL*	T>C	3´UTR miRNA binding site
**rs3217992**	*CDKN2B, ANRIL*	G>A	3´UTR miRNA binding site
