# Supplementary material for: Confirmation of involvement of new variants at CDKN2A/B in pediatric acute lymphoblastic leukemia susceptibility in the Spanish population
Source: PLoS One. 2017 May 8;12(5):e0177421. doi: 10.1371/journal.pone.0177421 (PMC5421813; doi:10.1371/journal.pone.0177421)
Supplement: S3 Table — SNPs with a MAF>10% are in bold. (PDF) [file pone.0177421.s004.pdf]

S3 Table: SNPs identified in 3´UTR region of *CDKN2A* and *CDKN2B*

| Gene | SNP | Alleles | MAF |
|---|---|---|---|
| CDKN2A | rs111532782 | G>C | 0.004 |
| | rs113798404 | - | - |
| | rs121913388 | C>G | 0.000008 |
| | rs142371511 | G>C | - |
| | rs145697272 | - | - |
| | rs182558871 | C>A | 0.001 |
| | rs190538376 | G>A | - |
| | rs200429615 | T>G | 0.001 |
| | rs201314211 | T>G | - |
| | rs34886500 | C>T | 0.0001 |
| | rs34968276 | C>A | 0.0001 |
| | rs36204273 | G>A | 0.0001 |
| | rs3731249 | G>A | 0.032 |
| | rs3731253 | C>G | - |
| | rs3731255 | C>G | - |
| | rs4987127 | G>A | - |
| | rs6413463 | T>A | - |
| | rs11552822 | - | - |
| | rs121913383 | - | - |
| | rs121913385 | - | - |
| | rs137854597 | G>A | 0.000008 |
| | rs137854599 | - | - |
| | rs181022755 | - | - |
| | rs200863613 | C>A | - |
| | rs3088440 | G>A | 0.078 |
| | rs45476696 | - | - |
| CDKN2B | **rs1063192** | T>C | 0.45 |
| | rs140726127 | A>G | - |
| | rs148421170 | C>T | 0.002 |
| | rs148786939 | T>C | - |
| | rs150924737 | T>A | 0.001 |
| | rs181736450 | C>A | - |
| | rs185130567 | T>C | 0.005 |
| | rs187514719 | A>G | 0.001 |
| | rs200344272 | G>A | - |
| | rs2285329 | T>C | - |
| | rs3217988 | G>A | - |
| | rs62637622 | G>A | - |
| | rs111751296 | - | - |
| | rs140430251 | - | - |
| | rs142570894 | C>T | 0.0003 |
| | rs144131923 | - | - |
| | rs183610933 | T>C | - |
| | rs187657501 | T>C | - |
| | rs3217983 | C>T | - |
| | rs3217990 | C>A | 0.002 |
| | **rs3217992** | G>A | 0.4 |

SNPs with a MAF>10% are in bold
