# Supplementary material for: Confirmation of involvement of new variants at CDKN2A/B in pediatric acute lymphoblastic leukemia susceptibility in the Spanish population
Source: PLoS One. 2017 May 8;12(5):e0177421. doi: 10.1371/journal.pone.0177421 (PMC5421813; doi:10.1371/journal.pone.0177421)
Supplement: S4 Table — SNPs, single nucleotide polymorphisms; PCR, polymerase chain reaction; RFLP, restriction fragment length polymorphism; bp, base pairs. (PDF) [file pone.0177421.s005.pdf]

S4 Table: Primers and PCR conditions for the amplification of rs3731249 in *CDKN2A*

SNP	Primer Sequences (5'- 3')	Genotype method	Restriction Enzyme	Fragment length (bp) according to the genotype
**rs3731249**	F1: GTGGACCTGGCTGAGGAG<br>R1: TCGGGATTATTTCCCATTTG	PCR-RFLP	*BstUI*	GG: 133, 73, 48, 29,21,2<br>AG: 181, 133, 73, 48, 29, 21, 2<br>AA: 181, 73, 48, 29, 21, 2

SNPs, single nucleotide polymorphisms; PCR, polymerase chain reaction; RFLP, restriction fragment length polymorphism; bp, base pairs.
